# Application of anterior debridement and reconstruction with anatomical screw-plate fixation for lumbosacral tuberculosis: A 2-year-plus follow-up

**DOI:** 10.1097/MD.0000000000007103

**Published:** 2017-06-30

**Authors:** Wen-Jun Wang, Wen-Kang Chen, Yi-Guo Yan, Nu-Zhao Yao, Cheng Wang

**Affiliations:** Department of Spine Surgery, the First Affiliated Hospital, University of South China, Hengyang, Hunan 421001, China.

**Keywords:** anatomical screw-plates, anterior approach, lumbosacral junction, spine reconstruction, tuberculosis

## Abstract

This study aimed to determine the efficacy and safety of anterior debridement and reconstruction with anatomical screw-plate fixation in patients with lumbosacral junction tuberculosis (TB).

A total of 48 patients (30 males and 18 females) diagnosed with lumbosacral junction TB were included in this study. All patients underwent surgery in our institution from January 2008 to July 2014, using anterior debridement and reconstruction with anatomical screw-plate. Outcome data were evaluated before and after surgery and included lumbosacral angle, Frankel classification, bone fusion, and visual analog scale (VAS) scores.

All patients were then followed up for an average of 49.4 months (range, 24–96 months). The mean lumbosacral angle improved from 8.36° ± 5.92° pre-operation to 22.38° ± 4.52° post-operation and 21.13° ± 3.73° during the final follow-up (both *P* < .05). Solid vertebral fusion was achieved in all patients after 7.6 months on average (range, 6–12 months). No severe complications appeared during operation and post-operation. Neurological performance and VAS scores were significantly improved compared with pre-operation (*P* < .05).

Following standard anti-TB chemotherapy, anterior debridement and reconstruction with anatomical screw-plate fixation may be a feasible and effective therapeutical option for lumbosacral junction TB. This procedure can result in satisfactory bone fusion and deformity correction, and effectively restore lumbosacral junction stability.

## 1 Introduction

Tuberculosis (TB) is a chronic consumptive disease with weight loss that represents a significant threat for humanity. In fact, TB has already caused over 1 million deaths worldwide and there are 10 million new infection cases per year, especially in the developing countries like China and India.^[1,2]^ Tuberculous spondylitis represents approximately 2% of all TB cases and often causes the destruction of vertebral bodies and endplates, generating spinal deformity, compression of the spinal cord and the adjacent nerve roots.^[3]^ Lumbosacral junction TB only accounts for 2% to 3% of spinal TB.^[4]^ The treatment options of tuberculous spondylitis include conservative antituberculous (anti-TB) therapy and the administration of anti-TB drugs combined with surgery. In the early infectious stage, regular and sufficient chemotherapy plays a critical role in the treatment of lumbosacral TB. Advanced TB with severe destruction of the lumbosacral junction, advanced neurological deficit, and loss of a normal lumbar lordosis with altered lumbosacral biomechanics urgently requires surgical approaches to relieve severe back pain, improve the neurological functions, and reconstruct the spine stability.^[5]^

Presently, various surgical debridement and fusion have been introduced for the treatment of lumbosacral TB, but the surgical strategies concerning the lumbosacral junction remain difficult and controversial. The therapeutical goals of these procedures are to eradicate infection, relieve nerve compression, prevent or correct deformity, and reconstruct spinal stability. However, the surgical treatment of patients with lumbosacral junction TB remains a clinical challenge because of the complex anatomy, the unique biomechanics, and the difficult fixation in the surrounding diseased bones of the affected region. One-stage posterior surgery has been applied for the treatment for lumbosacral TB, which can provide a stronger fixation, promote graft incorporation and fusion, and prevent graft slippage, allow early mobilization, and rehabilitation.^[6,7]^ However, posterior approach will damage the posterior osteoligamentous complex, leading to an increased instability of the spine. Moreover, some damaged tissue and abscess, which are confined to the anterior vertebrae, may not be removed.^[8]^ A further anteroposterior approach allowing to fix the lumbosacral segments can also provide satisfactory results in patients with TB spondylitis. Of note, anterior fixation devices for lumbosacral region can also be achieved with similar outcomes as the posterior debridement and bone grafting, and the mean operative time, blood loss, and hospitalization period are less than with anteroposterior approach.^[9,10]^ However, only few studies are available on the anterior debridement and reconstruction with anatomical screw-plate fixation to be adopted for a better clinical outcome in patients with advanced lumbosacral TB with cord compression or progressive deformity. In this study, we treated 48 patients with lumbosacral junction TB using anterior debridement and reconstruction with anatomical screw-plate fixation and evaluated the effectiveness and safety of this procedure.

## 2 Materials and methods

### 2.1 Basic information

This was a retrospective, clinical study. From January 2008 to July 2014, this study analyzed outcomes from 48 patients diagnosed with lumbosacral junction TB. The male-to-female ratio was 5:3 with a mean age of 49 years (range, 18–85 years). For each patient, the diagnosis was based on typical symptoms, laboratory results such as erythrocyte sedimentation rate (ESR), anemia, hypoproteinemia, and imaging examination findings such as x-ray, computed tomography (CT), and magnetic resonance imaging (MRI). Intervertebral space collapse, vertebrae destruction, paravertebral and/or prevertebral abscess, and spinal cord or nerve root compression owing to abscess or bony debris were revealed by imaging examination. All patients showed significant constitutional symptoms such as weight loss, fever with sweats, severe low back pain, and/or radiation pain in the lower limb. Patients presented with these symptoms for 6.4 months on average (range, 2–12 months). Among the 48 patients, 5 suffered from radiation pain in the lower limb and 47 reported severe low back pain. No patients were HIV-positive. The mean ESR was 38.45 mm/h (range, 28–110 mm/h). The imaging examination showed that the lesions were all located between the L4 and S2 segments. In addition, 27 patients showed a presacral abscess and 22 presented with a paravertebral abscess. The neurological assessments were based on the Frankel Classification^[11]^ and showed a spinal cord injury of grade B in 1 case, grade C in 26 cases, and grade D in 21 cases. The preoperative pain was evaluated using a visual analog scale (VAS) score.

### 2.2 Inclusion and exclusion criteria

Patients who presented with any of the following conditions were included in the study: intolerable back and/or radicular pain, progression of neurological deficit, conservative therapy is not effective, lumbosacral segment instability and deformity, large and sticky presacral and/or paravertebral abscess. In contrast, patients who met any of the following conditions were excluded: previous pelvic or lumbosacral surgery; lumbosacral deformity induced by any other disease, such as adolescent scoliosis or ankylosing spondylitis; lesions confined to the posterior vertebral column.

### 2.3 Preoperative procedure

Three-dimensional computed tomographic angiography (3D-CTA) of the iliac great blood vessels was performed preoperatively. All patients received standard anti-TB drugs via the (isoniazid [H] rifampin [R] ethambutol [E] pyrazinamide [Z]) (HREZ) (isoniazid [INH], rifampin [RFP], ethambutol [EMB], and pyrazinamide [PZA]) chemotherapy regimen for >2 weeks before the operation. The HREZ dosing consists of 300 mg/day INH, 450 mg/day RFP, 750 mg/day PZA, and 750 mg/day EMB. Moreover, patients received good general supportive care including bed rest and hyperalimentation before surgery. The latter was carried out when anemia and hypoproteinemia approximately returned to their normal level and when ESR was significantly decreased.

### 2.4 Surgical procedure

**Figure 1:**
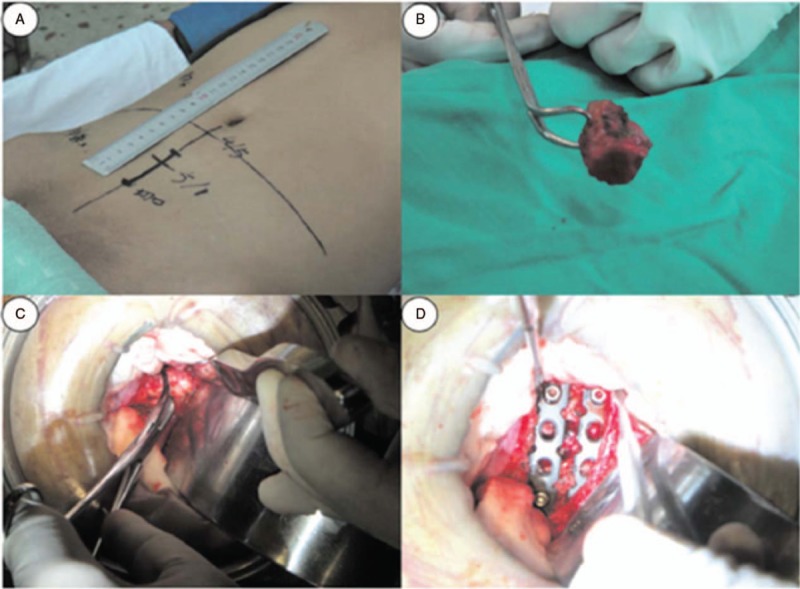
Intraoperative images of a 30-year-old male. (A) A 5-cm median incision by the linea alba was made around the lesion level through the skin and along the symphysis pubis to the umbilicus. (B and C) Autologous iliac bone was packed to the prepared bone groove of the L5–S1 level. (D) Two anterior reconstructive anatomical screw-plates of suitable length were fixed anteriorly at the L5–S1 level.

**Figure 2:**
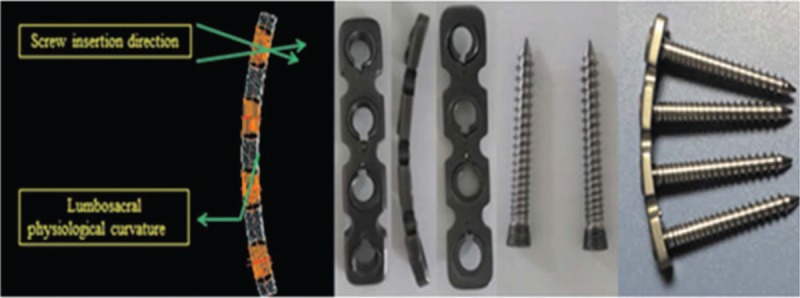
The anatomical screw-plate system manufactured by the Zhejiang Guangci Medical Instrument Company presented the following advantages: the direction of the screw insertion is adjustable; the screw-plate system can be suited to the lumbosacral physiological curvature; the system allows long segment fixation; the screw-plate system had anti-back and self-lock features.

After general anesthesia, patients were placed in the Trendelenburg position while monitoring somatosensory-evoked potential. The lesion site of lumbosacral TB was exposed via the anterior route (Fig. 1A). When the sacral promontory was validated by x-ray, the prevertebral fascia, the abdominal aorta, the inferior venacava, and its bifurcations were exposed. Then, the sacral artery and vein were ligated. Next, 4 long Steinmann pins with hose protection were inserted into the upper and lower vertebra before debridement. The use of wet gauze was necessary to prevent contamination of normal tissues from *Mycobacterium tuberculosis* and pull carefully apart the iliac vessels of both sides. We eliminated pus, sequestrum of the destructed vertebral bodies, cheesy necrotic tissues, lesional adhesion tissues, granulation tissues, and the affected disc with various curettes and rongeurs until bleeding bony surfaces were reached. Hydrogen peroxide and physiological saline were used to directly irrigate the lesion site until the wound was clear. Two appropriately sized tricortical autologous iliac bones with adequate autologous or allograft cancellous bone were tightly packed to the prepared bone groove of the lumbosacral spine (Fig. 1B, C). After measuring the length of the bone defect, 2 screw-plates of suitable anatomical length (Fig. 2) were fixed on the anterior part of the lumbosacral vertebrae (Fig. 1D). Alternatively, the pre-bent anatomical screw-plates could also be installed on theL4-S2 segments by traversing the bottom of the iliac vessels. Finally, irrigation of wound, intromission of streptomycin powders (1.0 g) and INH (0.3 g), drainage, and incision sutures were performed. All debrided specimens were analyzed for bacterial culture and histopathologic examination.

### 2.5 Postoperative procedure

The surgical drain was removed when the volume of fluid was <30 mL/24 hours. Intravenous antibiotics were administered to prevent infection. After lying supine on average for a week after the operation, patients were encouraged to start walking with the assistance of a thoracolumbar orthosis. All patients received the aforementioned 4-drug HREZ chemotherapy orally for 12 to 18 months after surgery. Bed rest and healthy nutrition were recommended to all patients. The plastic orthosis was removed when patients showed callus formation according to imaging data. All patients were clinically and radiologically examined after 1, 3, 6, and 12 months post-operation and then every year.

### 2.6 Follow-up period and statistical analysis

The average follow-up period was 49.4 months (ranging between 24 and 96 months). The following outcome measures were recorded before and after surgery, and during the follow-up period: the lumbosacral angle formed by 2 lines: one running along the posterior border of the first normal upper vertebra and the other one running through the posterior margin of S1^[12]^; the neurological status determined according to the Frankel Classification; the ESR; the VAS score, which was collected from all patients as a clinical outcome assessment; the degree of bone fusion, which was assessed by the Brantigan-Steffee classification^[13]^ to assess the trabecular bone formation: A, B, and C deemed no union, whereas D and E were considered as satisfactory fusion. Independent *t* tests were used to compare lumbosacral angle, ESR, and VAS scores before and after surgery, and/or during the follow-up period using SPSS 18.0 software (SPSS Inc, Chicago, IL). Data that did not follow a normal distribution were analyzed using the Wilcoxon rank-sum test. *P* values <.05 were deemed significant. All results were expressed as mean ± standard deviation.

## 3 Results

### 3.1 Operative results

All patients underwent surgery using anterior debridement, interbody fusion with anterior reconstructive anatomical screw-plates. The results are summarized in Table 1. The mean operation time was 131.9 minutes. The mean blood loss was 351.9 mL (range, 120–1000 mL). Average hospitalization was 8 days (range, 7–14 days). Patients had no recurrence at the end of the follow-up period.

**Table 1:**
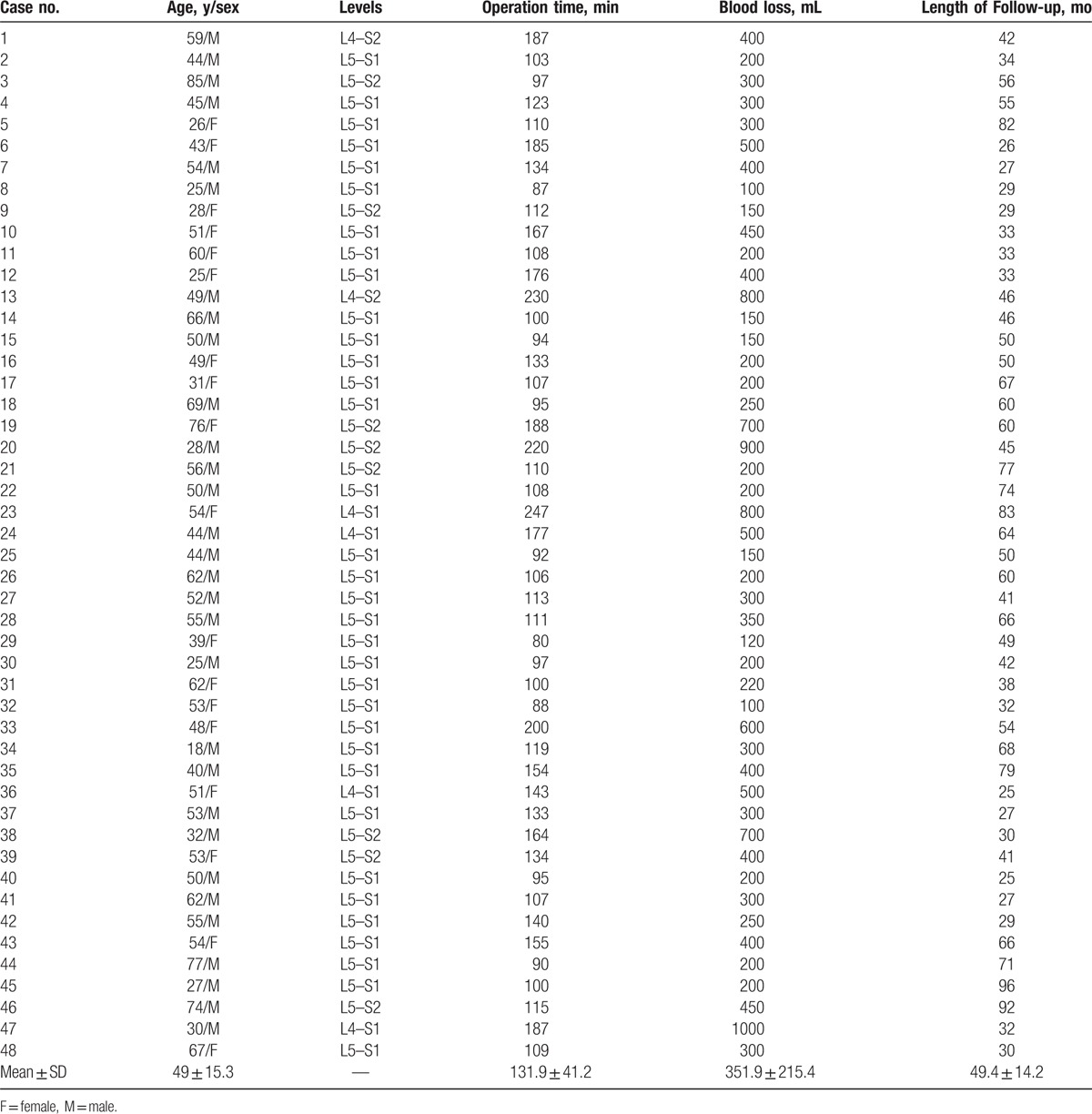
Summary of the patient demographics, operative information, and follow-up period.

### 3.2 Radiographic and laboratory results

**Figure 3:**
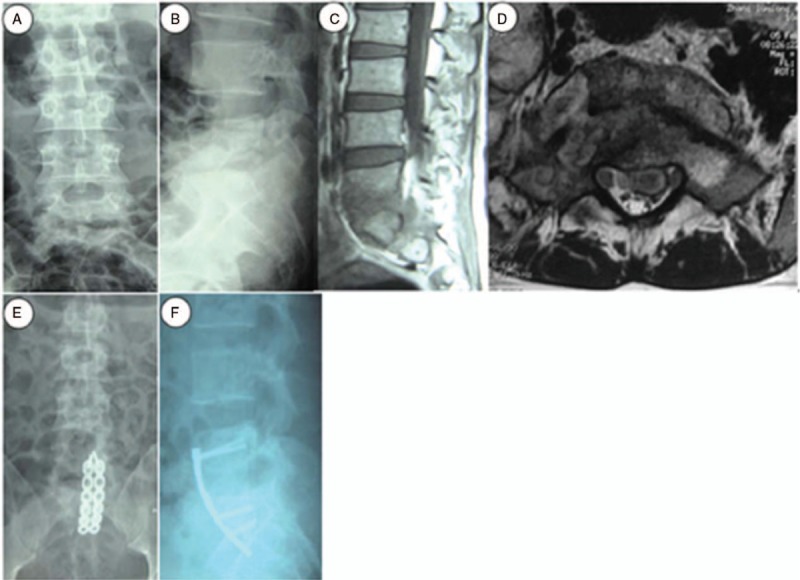
A 28-year-old woman underwent anterior debridement and reconstruction with anatomical screw-plate fixation for L5–S2 TB in our hospital. Preoperative, posteroanterior (A) and lateral (B) plain radiographs showed the L5–S2 vertebral damage with a narrowed intervertebral space and a decreased lumbosacral angle. The sagittal (C) and coronal (D) MRI views showed bony destruction from the L5 to S2 vertebrae, with prevertebral and paravertebral abscesses compressing the neural elements. Anteroposterior (E) and lateral (F) immediate postoperative radiographs showed the anterior column reconstruction with two 5.6-cm-long anatomical screw-plates across L5–S2.

**Figure 4:**
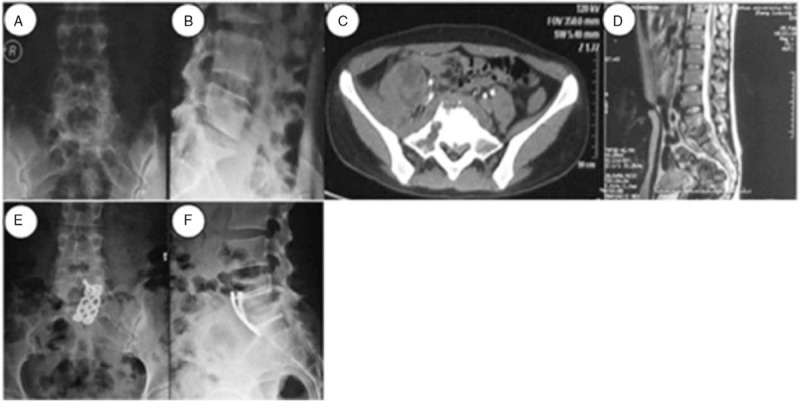
A 26-year-old woman was diagnosed with L5-S1TB and underwent anterior debridement and reconstruction with anatomical screw-plate fixation. Before surgery, posteroanterior (A) and lateral (B) plain radiographs showed the L5–S1 vertebral damage with a narrowed intervertebral space and a decreased lumbosacral angle. The CT (C) and MRI (D) scans showed the destruction of L5 and S1 vertebrae, with a cold abscess compressing the neural elements. During the last follow-up visit, the x-ray images (E and F) showed a complete correction of the lumbosacral angle and a solid fusion.

All patients were achieved satisfactory bone union 7.8 months on average after surgery (range, 4–12 months). At the end of the follow-up period, 26 patients reached the level E and 22 patients the level D of the Brantigan-Steffee classification. The average level of ESR was 3.24 mm/h (range, 0–28 mm/h) (Table 2). Compared with its preoperative value, the lumbosacral angle significantly improved after surgery and during the final visit of the follow-up period (Table 2; *P* < .05). Furthermore, as shown in Figures 3 and 4, we selected 2 typical patients suffering with serious lumosacral TB to display the pre- and postoperative images, respectively.

**Table 2:**
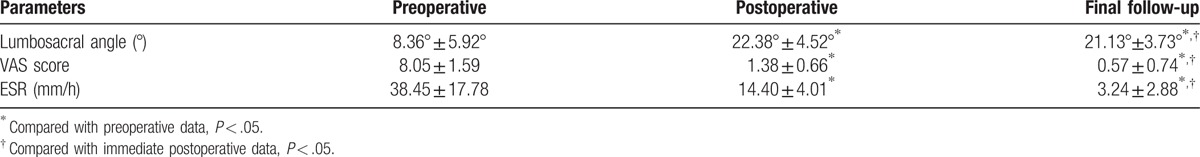
Mean values of the clinical and radiological parameters measured before surgery, 1 year after surgery, and at the final follow-up visit.

### 3.3 Clinical outcomes

Compared with its preoperative value, the VAS score significantly improved after surgery and during the final visit of the follow-up period (Table 2; *P* < .001). During the final visit, the neurological status of the 48 patients presenting with preoperative neurological deficits (according to the Frankel classification) had evolved as follows: 1 patient with grade B recovered to grade C; 26 patients with grade C recovered either to grade D (n = 4) or grade E (n = 22); 21 patients with grade D recovered to grade E (Table 3).

**Table 3:**
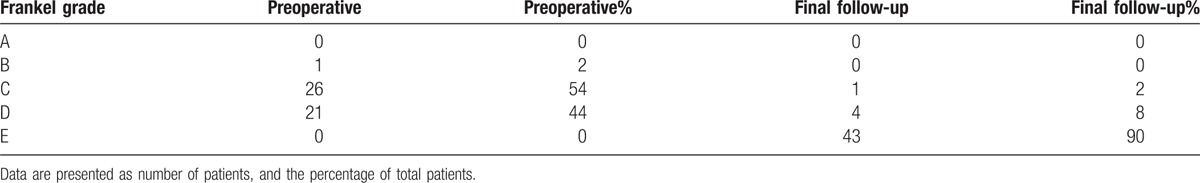
Neurological recovery according to the Frankel grade.

### 3.4 Complications

Of the 48 patients included in the present study, 4 patients presented with drug-resistant TB and 9 patients showed abnormal liver function and gastrointestinal tract reaction. After changing the chemotherapy regimen, these patients gradually recovered. Four patients presented donor site pain >1 year after surgery, successfully treated with <3 sets of superior cluneal nerve blocks. None of these patients revealed large blood vessels, bowels, ureter, bladder, or nerves injuries during surgery. Eight patients suffered from transient abdominal distention, and 6 had urinary retention, which recovered spontaneously after 1 week without any special treatment. Three patients demonstrated persistent pain after surgery and underwent a treatment with analgesics. On average, pain significantly decreased 5 days after surgery. Six patients presented with hypokalemia, and 3 showed hyponatremia. After receiving intravenous rehydration, these water-electrolyte disorders were corrected. One patient developed wound infections. This patient quickly recovered owing to the successful application of debridement and a systemic antibiotic. During the long-term follow-up period, no severe complications, such as tuberculous peritonitis, ileus, erectile dysfunction, or retrograde ejaculation, and no implants or instrumentation-related stabilization problems were observed.

## 4 Discussion

The development of spinal TB in the lumbosacral junction, located between the lumbar lordosis and the sacral kyphosis, is relatively rare. However, the stability of this segment is important because of the high mechanical load that it bears. In patients with severe lumbosacral junction TB, the destruction of the vertebral bodies and the intervertebral discs could dislocate the lumbosacral joint, thus resulting in joint capsule laxity. Hence, lumbosacral junction TB easily contributes to spinal instability and nerve damage. The surgical treatment of lumbosacral junction TB aims to eradicate the site of infection, prevent spinal deformity, relieve pain, and reconstruct the stability of the lumbosacral segments. Anti-TB chemotherapy can effectively inactivate TB, but patients will then have a higher incidence of low back pain owing to the risk of spinal instability or the progression of neurological deficit.^[14]^ In addition, long-term anti-TB drug therapy is known to cause various side effects, such as liver dysfunction, skin reactions, and gastrointestinal and neurological disorders. In our study, 9 patients demonstrated drug complications after surgery. To date, studies have reported different surgical indications for the treatment of lumbosacral junction TB, such as a persistent back and/or radicular pain that is resistant to conservative therapy, a persistent neurological deficit, a significant kyphosis, or a progressive deformity.^[15]^ In our study, patients presented with prevertebral and/or paravertebral abscess, severe back pain along with radiculopathy, advanced neurological deficits, or spinal instability.

In 1960, Hodgson et al first reported satisfactory results through radical anterior debridement and fusion with a strut graft in patients with spinal TB.^[16]^ The current anterior approach for lumbosacral TB includes both the extraperitoneal and transperitoneal approaches. Many researchers reported that patients with lumbosacral TB obtained encouraging deformity correction rate and an improvement of their neurological status with an anterior approach. By comparing anterior and posterior instrumentation in the correction of kyphotic deformity of patients with tuberculous spondylitis, Benli et al^[17]^ showed that anterior instrumentation was as effective as posterior instrumentation. Also, Li et al^[18]^ reported that 43 patients treated with extraperitoneal anterior approach could effectively achieve deformity correction and restore lumbosacral junction stability. Results of our research are consistent with these statements. In our study, the lumbosacral angle improved on average, from 8.36° ± 5.92° before surgery to 22.38° ± 4.52° (*P* < .05) after surgery and 21.13° ± 3.73° at the end of the follow-up period (*P* < .05). The final follow-up examinations revealed that half of the patients (n = 24) showed a complete neurological recovery. There were statistically significant improvements before and after treatment regarding to lumbosacral angle, neurological status, and ESR. All patients showed a solid bone fusion 6 to 12 months after surgery. There were no apparent pseudarthroses and implant failures.

The common advantages of the anterior approach are as follows: there is no destruction of the posterior structures and a single incision by the linea alba can significantly reduce the muscle damage; an extensive discectomy and several implants can restore disc height and correct the segmental lordosis; moreover, this approach can also offer more favorable biomechanical conditions for bone fusion; a direct access to the lesion and a direct reconstruction can facilitate the focal debridement and nerve decompression; this approach offers the best view of disc space compared with other procedures, which can decrease the surgical damage to blood vessels, ureters, and nerves; if the psoas or iliac abscess was flowed, this procedure allows the surgeon to remove it directly and thereby effectively prevent TB recurrence; in contrast, this may constitute a restriction for the 1-stage posterior approach^[19]^; (6) the short operative time and the short hospital stay can reduce the financial burden of patients.

Up to now, various anterior anatomical plates have been applied and evaluated clinically for anterior lumbosacral fixation. The biomechanical and clinical assessments have shown that using anterior plates could improve the stability of the lumbosacral junction fixation. By direct biomechanical comparisons of the anterior lumbosacral screw-plate fixation with the stand-alone interbody cages, Gerber et al^[20]^ revealed that anterior screw-palte fixation resulted in significantly less range of motion and more stiffness. In addition, Beaubien et al^[21]^ conducted a biomechanical comparison between anterior lumbar interbody fusion (ALIF) with anterior tension band plates and posterior pedicle screws, and showed that anterior tension band plates add significant ALIF) stability, although they are not as rigid as pedicle screws. Furthermore, by comparing the anteroposterior approach with pedicle screws fixation with anterior approach with lumbosacral vertebrae plates fixation performed on lumbosacral TB cases, He et al concluded that anterior plates can be served as a available single-method alternative to supplemental posterior pedicle screw fixation.^[10]^ In this study, we improved the anatomical screw-plate system on the lumbosacral spine site, which has the following advantages: the direction of the screw insertion is adjustable; the screw-plate system can be suited to the lumbosacral physiological curvature; the system allows long segment fixation; the screw-plate system has anti-back and self-lock features.

However, various complications associated with the anterior approach of the lumbosacral lesions have been described in the literature, including vascular complications, bladder, nerve, ureter, and bowel injuries, as well as abdominal wall complications.^[22]^ Among them, the most potentially dramatic complications that must be considered are vascular complications. Thus, some authors advised against the application of anterior implants at the lumbosacral segments owing to the iliac vessels crossing the vertebral bodies.^[23]^ To our knowledge, the lumbosacral junction is typically exposed by clearing the prevertebral vascular structures. The left common iliac vessels coursed obliquely across the anterior aspect of the L5 vertebrae, thereby traversing portions of L5–S1 disc spaces. Most causes of vein injury come from the laceration of the venovertebral veins or the left common iliac vein during mobilization.^[22,24]^ Thus, it is important to evaluate the anatomic configuration of the prevertebral vessels before surgery, which can significantly reduce the risk of perioperative vascular injuries and unnecessary exposure in the lumbosacral junction area. In our study, all patients were scanned with a 3D-CTA of the iliac great blood vessels to prevent this complication preoperatively, and no patients showed vascular complications intraoperatively. Although anterior approach with lumbosacral screw-plate fixation can prevent the implant from dislocating and sinking into the disc space owing to its excellent supportive ability, the operation is technically demanding and should only be used by surgeons whose extensive experience can improve success rate.

The findings obtained in the present study are encouraging. At the end of follow-up period, 44 patients had returned to fulltime employment. All patients demonstrated clinical healing of TB without recurrence and reactivation. However, the individual evaluation of each patient with lumbosacral junction TB must be considered in the clinical application of this surgical procedure. This approach recommends to be used in the following conditions: lesions mainly confined to the anterior and middle column of lumbosacral vertebrae, severe lumbosacral destruction combined with large and sticky psoas or iliac abscesses, abscess or bony debris resulting in coccygeal nerve or nerve root of lumbosacral vertebrae compression.

This retrospective observational study has some limitations. First, the main drawback of our study is a small sample size. Second, all the patients of this study presented with a disease affecting <4 vertebraes. Third, a long-term follow-up is lacking in this study. Therefore, future research should consider a larger number of patients and a longer follow-up period.

## 5 Conclusion

To date, an established and definitive classification system is lacking for the therapy of lumbosacral junction TB. The results of this study demonstrate that anterior debridement and reconstruction with anatomical screw-plate fixation is an available treatment method for lumbosacral junction TB. Moreover, this method can provide satisfactory bone union and stabilize the lumbosacral junction. Furthermore, with the recent progress of the 3D printing technology, our center will use individualized anatomical screw-plates for this surgery.

## Acknowledgments

The authors gratefully acknowledge the financial support from the National Natural Science Foundation of China (31570946), and the Natural Science Foundation in Hunan Province, China (2015JJ5003).
